# P02.30. Assessment of hypnotizability in clincal research: development, reliability, and validation of the Elkins Hynotizability Scale

**DOI:** 10.1186/1472-6882-12-S1-P86

**Published:** 2012-06-12

**Authors:** G Elkins, W Fisher, A Johnson

**Affiliations:** 1 Baylor University, Waco, USA

## Purpose

Hypnotherapy has been shown to be an effective treatment for some conditions such as pain, anxiety, chemotherapy side effects, and hot flashes. Measurement of hypnotizability is essential for research. There are a number of scales that have been developed to measure hypnotizbility in clincal research. However, the currently available scales suffer from a number of important shortcomings such as time for administration, validity concerns, and item difficulty. As a result, hypnotizability is rarely measured in clinical work and research has been limited. The purpose of this study was to develop a new scale to measure hypnotizability that met the following criteria: (1) administration in 30 minutes or less; (2) correlation of .80 or better with the Stanford Hypnotic Susceptibility Scale; and (3) improved item selection.

## Methods

A 12 item hypnotizability scale (Elkins Hypnotizability Scale; EHS) was developed with the wording for each item based upon the clinical experience of the Principal Investigator from administration of the EHS in clinical work and receiving feedback from patients and expert reviewers.

## Results

The average time for administration was 27 minutes. Test-retest reliability was r = .93. The correlation between the EHS and the Stanford Hypnotic Susceptibility Scale-Form C was r = .91.

## Conclusion

Results support the development of an improved scale to measure hypnotizability. The results demonstrated that the scale can be administered within 30 minutes (as compared to 75 minutes for the Stanford Hypnotic Susceptibility Scale-Form C). In addition, the scale demonstrated strong reliability. The correlation between the EHS and Stanford Hypnotic Susceptibility Scale-Form C was also strong and significant, suggesting that the EHS measures the same domain of hypnotic responding and is therefore likely to be a valid measure. The present study reports on initial scale development, and the utility of the EHS in clinical research is presently being determined.

